# Mechanisms underlying centriole stability

**DOI:** 10.1016/j.jbc.2025.110869

**Published:** 2025-10-28

**Authors:** Erica Biven, Jennifer T. Wang

**Affiliations:** Department of Biology, Washington University in St Louis, St. Louis, Missouri, USA

**Keywords:** centrosomes, cilia, basal bodies, centrioles, microtubules, cartwheel, inner scaffold, pericentriolar material, A-C linker, centriole loss, centriole elimination, programmed centriole loss, protein turnover

## Abstract

Centrioles and basal bodies are conserved, highly stable microtubule-based supramolecular structures. Together with the pericentriolar material, centrioles form the major microtubule organizing center of animal cells, known as the centrosome. Centrioles can be structurally altered to form basal bodies, which template the formation of cilia, specialized protrusions from eukaryotic cell membranes involved in cell signaling, motility, and fluid movement. Centrioles and basal bodies are normally extremely stable: They can withstand numerous rounds of cell division and are resistant to cold treatment, microtubule depolymerizing drugs, and other chemical treatments that disassemble cytoplasmic microtubules. Here, we discuss recent advances in our understanding of how centrosomal substructures, which include the centriolar microtubule walls, cartwheel, inner scaffold, and pericentriolar material, contribute to the long-term stability of centrioles and basal bodies. We also review how the regulated loss of these substructures can trigger centriole elimination in multiple tissues and organisms.

## Centriole architecture and formation

Centrioles and basal bodies are among the largest supramolecular structures found in eukaryotic cells. At ∼250 nm in width and ∼500 nm in length, these conserved gigadalton-sized organelles are larger than nuclear pores (120 nm in diameter) (1, 2) or ribosomes (∼25 nm in each direction) (3). Two centrioles, along with the pericentriolar material (PCM), a protein matrix that concentrates proteins and nucleates microtubules, form a centrosome. Centrosomes are the major microtubule organizing center of animal cells. They nucleate and anchor microtubules, serve as the poles of the bipolar mitotic spindle, nucleate actin, establish cell polarity, control cell cycle progression, and regulate the immunological synapse (4, 5, 6, 7, 8). A centriole can be remodeled into a basal body that templates the formation of a cilium, which projects from the cell surface for cell signaling, motility, and fluid movement. Centrioles and basal bodies are found in all major branches of the eukaryotic tree of life and were likely present in the last eukaryotic common ancestor (9, 10). Structural, functional, or numerical defects in centrosomes and cilia are associated with a variety of disease states, including microcephaly, primordial dwarfism, cancers, Trisomy 21, and ciliopathies (7, 11, 12, 13, 14).

**Figure 1:**
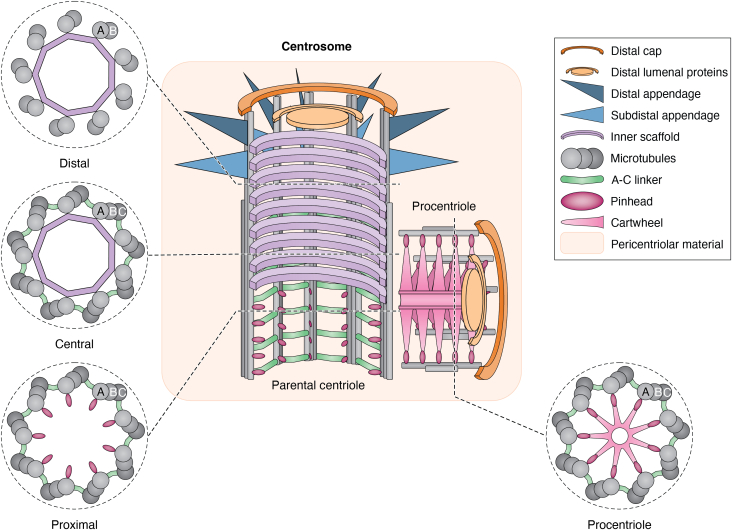
**The architecture of the centrosome.** The centrosome is formed of two centrioles surrounded by pericentriolar material (tan rectangle). Centrioles are cut in half to reveal inner structures, and cross-sections are shown at the indicated positions. The parental centriole is vertical and templates the formation of a procentriole at a right angle.

Centriole structure is highly conserved and intimately linked to function. In most species, the centriole wall is composed of linked, “compound” microtubules arranged in ninefold symmetry: three linked microtubules form the triplets at the proximal end, and two linked microtubules form the doublet microtubules at the distal end (Fig. 1). Compound microtubules are only found in centrioles and cilia and are conserved in almost all organisms with these organelles (15). This indicates that their unique microtubule arrangement is crucial for the organization and function of centrosomes and cilia. The three linked microtubules in the triplets are termed the A-, B-, and C-tubules: the A-tubule is a complete microtubule of 13 protofilaments, and the B- and C-tubules are partial tubules of 10 protofilaments each, which share walls with the adjacent microtubules. The A- and B-tubules elongate to form the linked, “doublet” microtubules at the distal end. During ciliogenesis, the A- and B-tubules further elongate to form the microtubule core of the cilium, known as the axoneme.

Centrioles exhibit proximal-distal polarity, featuring non-microtubule elements that associate with the microtubule wall in each region. We refer to these elements as "centrosome substructures." These include the cartwheel at the proximal end of the centriole, primarily formed by the protein SASS6, which self-assembles into a hub and spokes that imparts ninefold symmetry and the stereotypical ∼250 nm width upon the centriole (16, 17). The cartwheel is attached to the microtubule walls through the pinhead, whose composition is unknown. The A-C linker is an additional substructure that connects adjacent triplet microtubules in the proximal end (18). Within the central core region, the helical inner scaffold substructure connects the triplet microtubules (19, 20, 21, 22, 23, 24, 25). At the distal end, several different protein complexes are involved in regulating centriole elongation, enabling ciliogenesis, and maintaining centriole structure. The PCM surrounds two centrioles to form the centrosome. Surrounding the centrosome, membrane-less organelles known as centriolar satellites concentrate proteins and RNAs to regulate centrosomes and cilia (26, 27, 28).

**Figure 2:**
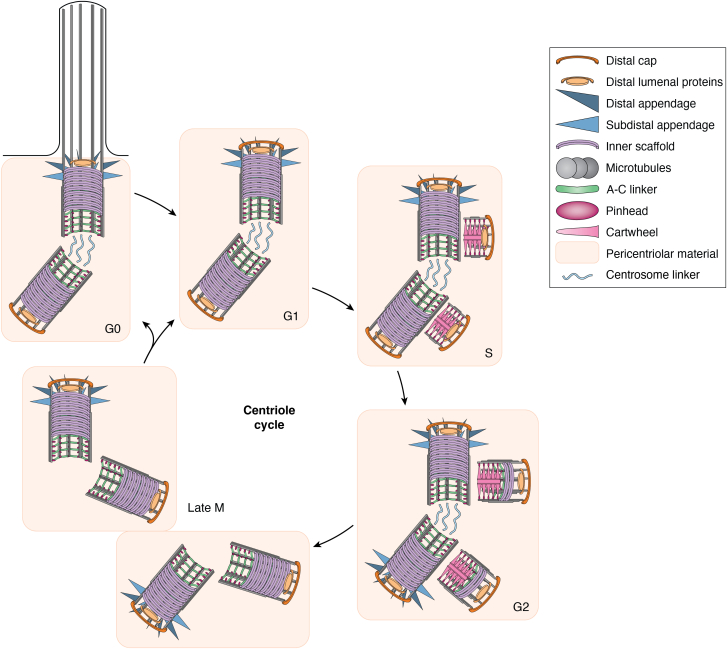
**Centrosome structure and number through the cell cycle.** In S phase, each parental centriole templates the formation of a procentriole. In G2 phase, the procentrioles continue to grow and elongate, and both parental centrioles have acquired appendages. In mitosis, the linker between the parental centrioles dissolves, resulting in two centrosomes segregated on opposite poles of the mitotic spindle. Each daughter cell receives one centrosome, containing a parental centriole and a daughter centriole. The linker between those centrioles forms again in G1. Upon exit from the cell cycle into G0, the grandmother centriole with appendages can template the formation of a cilium.

In cycling mammalian cells, centriole number and structure are tightly regulated by the cell cycle (Fig. 2). In G1 phase, cells have two centrioles. In S phase, each preexisting parental centriole directs the formation of a new procentriole, leading to cells with 2 parental centrioles and 2 procentrioles. Procentriole formation is initiated in S phase by the kinase PLK4, resulting in the formation of the cartwheel. In G2, procentrioles elongate and build the inner scaffold (19, 25). In mitosis, centrioles recruit additional PCM to nucleate microtubules for mitotic assembly. During mitosis, the cartwheel is lost from procentrioles, which continue to elongate and start to recruit pericentriolar material in a process known as centriole-to-centrosome conversion (29, 30, 31, 32, 33, 34, 35). Centrioles are segregated on the poles of the mitotic spindle, resulting in each daughter cell receiving two centrioles—one older parental centriole and one younger centriole, both surrounded by pericentriolar material.

As the younger centriole matures in the subsequent cell cycle, it acquires appendages at its distal end (36, 37): the distal appendages that attach to membranes for ciliogenesis and the subdistal appendages that anchor microtubules to the centrosome. When cells enter G0 phase, the maturation of the older of the two centrioles into a basal body allows the generation of a cilium through a complex process that involves membrane attachment to the distal appendages, loss of the distal end cap, and elongation of the centriolar doublet microtubules to form the ciliary axoneme (7).

Some differentiated cells, including olfactory sensory neurons, placental trophoblast cells, and multiciliated cells of the respiratory tract, oviduct, and brain ventricles, increase their number of centrioles and basal bodies beyond the typical two to four centrioles found in cycling cells. For additional information about centriole architecture and formation in other tissues, the reader is referred to several excellent recent reviews (7, 38, 39, 40, 41, 42).

## Overview of centriole stability and loss

Centrioles have traditionally been regarded as exceptionally stable organelles. They are inherited through numerous rounds of cell division; in *Caenorhabditis elegans,* the centrioles contributed by sperm to the newly fertilized embryo persist up to the 550-cell stage (43). Centrioles can withstand extreme conditions, including microtubule depolymerizing drugs, cold treatment, and high salt (44, 45, 46, 47). Unlike cytoplasmic microtubules, which undergo dynamic instability, the microtubule walls of mature centrioles experience little turnover of tubulin (46). In addition, many structural centriolar proteins are stably incorporated on the scale of minutes to hours, including proteins of the cartwheel (SASS6), inner scaffold (POC5, CETN2, CCDC15, and POC1B), centriole lumen (PPP1R35), and A-C linker (CCDC77, WDR67, and MIIP) (19, 22, 48, 49, 50, 51, 52). By contrast, cytoplasmic microtubules are highly dynamic, with half-lives ranging from 5 minutes to an hour (53), and proteins of other organelles, such as the Golgi, diffuse on the timescale of minutes (54).

Despite their apparent stability, centrioles can be lost from cells during development, in disease, or through genetic manipulation. Here, we define centriole loss as the complete absence of centrioles within a cell. Some cases of centriole loss are preceded by centriole fragmentation, which we define as defects in centriole structure, such as broken microtubule walls or incomplete centriole architecture. By contrast, centrosome fragmentation refers to the distancing of centrioles from each other within a centrosome, or loss or relocalization of the pericentriolar material surrounding centrioles. In most cases of centrosome fragmentation, the structural status of the centriole has not been described.

Centriole loss has been reported during the development of a variety of organisms. When the single-celled eukaryote *Naegleria gruberi* transitions from a flagellated life form with two cilia to an acentriolar ameboid form, its centrioles are degraded in lysosomes and proteasomes, and are also eliminated from cells through extracellular shedding (55). In multicellular organisms, most sexually reproducing animals eliminate their centrioles during oogenesis (56, 57, 58). Interestingly, the mechanisms are species-specific: in starfish, centrioles are asymmetrically segregated into the polar body and eliminated in the cytoplasm; in *Drosophila*, centriole stability is conferred by the pericentriolar material; in *C. elegans*, stability is conferred by a centriole inner structure known as the central scaffold (59, 60, 61, 62, 63, 64). In developing *Drosophila* eyes, structurally aberrant centrioles are found along with centriole loss (65). In the *Drosophila* salivary gland, centrioles are exocytosed from cells by non-muscle myosin II and macroautophagy (66). Centriole loss is likely even more widespread: in *C. elegans,* centrioles are eliminated from most cell lineages during embryogenesis (67). Such a comprehensive analysis has not been performed for other organisms.

Maintenance of centriole integrity and proper regulation of centriole loss are essential to cell viability and tissue development. Loss of centriole integrity in *C. elegans* embryos results in cell division defects and embryonic lethality (68, 69). Improper regulation of centriole loss is also detrimental to cells: failure to eliminate centrioles in *Drosophila* oocytes results in abnormal meiotic spindle structure and aborted embryonic development (59), while failure to eliminate centrioles in *Drosophila* salivary glands is associated with disrupted mitochondrial structure and impaired cellular respiration (66).

Defects in centriole number and structure have also been reported in multiple disease conditions. While centrosome amplification is frequent in many cancers (70), centrosome loss has been reported to correlate with prostate cancer progression (71). In cancer cell lines, centriole structural integrity is compromised, resulting in over-elongated and fragmented centrioles (72). The disease mutation causing hydrolethalus syndrome also results in centriole fragmentation, though centriole loss has not been reported (13).

What regulates centriole stability and loss? Over the course of weeks (approximately 10 or more cell divisions), inhibiting new procentriole formation could potentially lead to centriole loss in tissues. In cultured cell lines treated with a PLK4 inhibitor, procentrioles are not produced, but parental centrioles remain (73). After several rounds of cell division, the proportion of cells with parental centrioles diminishes, resulting in most cells ultimately lacking centrioles.

**Figure 3:**
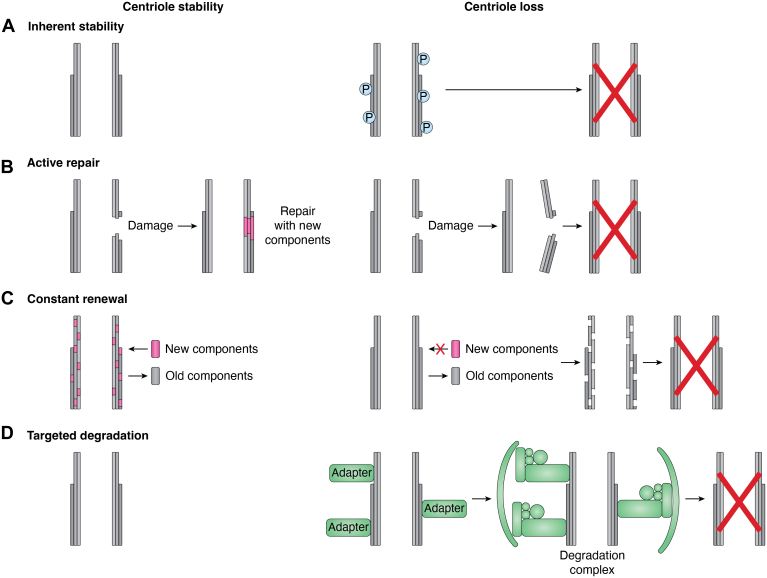
**Four non-mutually exclusive models for maintenance of centriole stability and mechanisms underlying centriole loss.***A*, inherent stability: Centrioles have inherent stability provided by their architecture. Disruptions to centriole structure, for example through mechanical stress or post-translational modifications of key proteins, (P), induce centriole loss. *B*, active repair: Centrioles are damaged and must be repaired with new components. If the damage is not repaired, centrioles may fragment, resulting in their loss. *C*, constant renewal: There is constant renewal of centriole components, such that old components are replaced with new components. If new components cannot be incorporated, the loss of old components will lead to centriole instability and loss. *D*, targeted degradation: Centriole loss is triggered by the binding of adapter proteins to the centriole, which then promote the degradation of centrioles through pathways such as autophagy.

However, many examples of centriole loss occur on a much shorter timescale, some within a single cell cycle. We propose four non-mutually exclusive models (Fig. 3). First, it is possible that centriole architecture has evolved to be inherently stable, and substructures anchor and fortify the microtubule walls (Fig. 3*A*). In this model, the proper formation of centriole architecture is essential for its stability. Disruptions to centriole architecture, for example, through mechanical stress or post-translational modifications of key proteins, may result in centriole fragmentation and loss. Second, cells may have evolved active mechanisms to repair damaged centrioles (Fig. 3*B*). Components with increased susceptibility to damage may require more frequent replacement. Inactivated repair mechanisms coupled with transcriptional downregulation of these key components may be responsible for centriole loss. Third, it is possible that despite their apparent stability, certain centriole components undergo constant renewal and turnover to prevent damage from accumulating (Fig. 3*C*). Studies using SILAC (stable isotope labelling by amino acids in cell culture) show that some centrosomal proteins do turn over (74). Results from FRAP (fluorescence recovery after photobleaching) experiments demonstrate that some centrosomal proteins exchange with the cytoplasmic pool (22, 48, 49, 50, 51). Protein turnover and exchange are important for centriole loss during *Drosophila* oogenesis and in *Drosophila* cell lines (42, 74, 75). Components with increased susceptibility to damage may undergo more frequent renewal, as “preventative maintenance”. Transcriptional downregulation of genes encoding these components would be sufficient to trigger centriole loss. Fourth, centrioles may be targeted for degradation in specific tissues by proteasomes, lysosomes, or autophagy (Fig. 3*D*). In this model, specific adapters recognize centriole components to target them for degradation. Centriolar satellites may play an important role in this process as they contain autophagy and proteasomal degradation factors, including the ATG8-family member GABARAP and the E3 ubiquitin ligases MIB1 and UBR5 (76, 77).

Some of these models have been proposed in specific contexts, and it is possible that several of these mechanisms are concurrently active. For example, loss of centriole integrity may precede targeted degradation. It is also possible that different organisms or cell types use different mechanisms to maintain their centrioles. Finally, centriole stability may be maintained differently in mature centrioles and procentrioles, and it is important to distinguish between defects in centriole formation *versus* stability after formation.

Until recently, investigating the structural integrity of centrioles was challenging due to their small size, which is at the resolution limit of light microscopy. However, with the advent of expansion microscopy and super-resolution microscopy, along with improved methods for identifying centriole components, many labs are now able to routinely examine centriole architecture (36, 78, 79, 80, 81, 82, 83). This has led to the identification of proteins responsible for maintaining structural integrity, revealing that these proteins localize to specific substructures of centrosomes: the microtubule walls, the cartwheel, the A-C linker, the centriole inner structure, and the pericentriolar material (Table 1). Disruption of these structures, whether it occurs during development or through experimental manipulation, destabilizes centrioles and causes their fragmentation and loss. Below, we review how these substructures contribute to centriole stability.

**Table 1:** Proteins and substructures involved in maintaining centriole stability

Protein	Substructure	Centriole/basal body fragmentation phenotype	Centriole/basal body loss phenotype	Associated diseases
TUBD1, TUBE1, TEDC1, TEDC2	Microtubule walls	Fragmentation in mitosis	Loss in mitosis	Unknown
*Chlamydomonas*, *Paramecium*, *Tetrahymena* Epsilon-tubulin, delta-tubulin	Microtubule walls	Broken triplets	Loss	See TUBD1, TUBE1, TEDC1, TEDC2
HYLS1	Microtubule walls	Fragmented at the distal end	Not reported	Hydrolethalus (161), Joubert syndrome (162)
Centrobin	Microtubule walls	Not reported	Overexpression leads to centriole destabilization	Unknown
*Drosophila* SAS-4	Microtubule walls	Not reported	Loss	See CPAP
CPAP	Microtubule walls	Short, narrow, or fragmented centrioles with splayed centriolar microtubules	Not reported	Primary microcephaly (163, 164), Seckel syndrome (165)
RTTN (rotating)	Proximal microtubule walls	Short centrioles	Loss in mitosis	Primary microcephaly (166, 167), developmental delay (167), primordial dwarfism (166), polymicrogyria (168), infantile dilated cardiomyopathy (169)
CEP350	Distal microtubules walls	Centriole fragmentation, with missing singlets, doublet and triplets in the distal end	Not reported	Unknown
*Drosophila* SAS-6	Cartwheel	Not reported	Loss in *Drosophila* cell lines	See SASS6
SASS6	Cartwheel	Long or fragmented centrioles in mouse embryonic stem cells	Loss in mouse embryonic stem cells	Over-expressed in cancers (170, 171), primary microcephaly (172)
CCDC77, WDR67, MIIP	A-C linker	Fragmentation of the centriole wall	Not reported	Unknown
WDR90	Inner scaffold	Centriole fragmentation, with splayed centriolar microtubules	Not reported	Unknown
*Tetrahymena* POC1	Inner scaffold	Missing triplet microtubules	Loss	See POC1A and POC1B
POC1A and POC1B	Inner scaffold	Short centrioles with broken centriole walls	Double knockout results in loss during mitosis	SOFT syndrome: short stature, onychodysplasia, facial dysmorphism, and hypotrichosis (173); cone-rod dystrophy (174)
*Tetrahymena* Centrin1 and Centrin2	Inner scaffold	Not reported	Loss	Unknown
CCDC15	Inner scaffold	Broken, wider, or shorter centriole walls	Loss of centrin foci upon prolonged mitotic arrest	Unknown
*C. elegans* SAS-1	*C. elegans* central tube	Centriole fragmentation	Premature centriole loss in oogenesis, centriole loss in sperm and embryos	Human C2CD3 is associated with oral-facial-digital syndrome and microcephaly (155, 175)
*C. elegans* SSNA-1	*C. elegans* central tube	Centriole fragmentation in embryos	Not reported	Unknown
MAP3K1	Distal centriole	Overexpression leads to centriole fragmentation	Not reported	46,XY gonadal dysgenesis (176, 177), Langerhans cell histiocytosis (178)
SFI1	Distal tip	Broken or fragmented walls	Not reported	Unknown
γ-TuRC and augmin	Centriole lumen and PCM	Short or broken centrioles	Loss of centriole distal ends upon prolonged mitotic arrest	Microcephaly (179), retinopathy (179), facial dysmorphism (179), cancers (180)
CEP295	PCM	Not reported	Loss in mitosis	Seckel-like syndrome: primary microcephaly, short stature, and craniofacial and digital abnormalities (181)
*Drosophila* ANA1	PCM	Not reported	Loss in *Drosophila* cell lines	See CEP295
*Drosophila* Polo	PCM	Not reported	Premature centriole loss in *Drosophila* oocytes	Cancer (182)

Summary of proteins and substructures in the text. Names refer to human proteins unless otherwise noted.

## The microtubule scaffold of the centriole

### Compound microtubules

The linked, compound “triplet” and “doublet” microtubules are highly conserved in organisms possessing centrioles, basal bodies, and cilia and are exclusively found in these organelles (15). Compound microtubules are composed of alpha-tubulin and beta-tubulin and are directly attached to the microtubule-binding proteins within centrioles (23, 24, 84, 85, 86, 87, 88, 89, 90). The mechanisms underlying compound microtubule formation are not understood and have been proposed to involve delta-tubulin, epsilon-tubulin, their associated proteins TEDC1 and TEDC2, and HYLS-1 (81, 91, 92, 93, 94).

In mammalian cells, loss of delta-tubulin (TUBD1), epsilon-tubulin (TUBE1), TEDC1, or TEDC2 leads to the formation of centrioles with just singlet microtubules (81, 93). These aberrant mutant centrioles fall apart starting in metaphase, with nearly complete loss of centrioles by G1 of the next cell cycle. Similarly, the loss of delta-tubulin or epsilon-tubulin from *Chlamydomonas*, *Paramecium*, and *Tetrahymena* is also associated with defects in the basal body triplet microtubules and their loss (91, 92, 95, 96, 97, 98). The molecular mechanisms by which these proteins function remain unclear. Delta-tubulin and epsilon-tubulin have additional roles in mouse male germ cell development, including stabilizing the kinetochore during meiosis and regulating the sperm manchette (99, 100).

Recently, HYLS-1 was reported to enable the formation of compound microtubules (94). HYLS-1 localizes to the centriole walls and is recruited during procentriole assembly. Centrioles in HYLS-1 null cells are fragmented at the distal end, supporting the idea that the compound microtubules are required for centriole integrity, though centriole loss has not been reported (13).

The stability of the compound microtubules likely relies on the proteins that directly attach to them. These include proteins of the inner scaffold, A-C linker, and pericentriolar material. They also include the proteins rotatin (RTTN), centrobin, and *Drosophila* SAS-4, which localize closely to the centriole walls (60, 101, 102, 103). RTTN null mutants have a similar phenotype to delta-tubulin and epsilon-tubulin null mutants, with centrioles that disassemble in mitosis (101). Over-expression of the tubulin-binding domain of centrobin leads to centriole destabilization, perhaps through competition with endogenous centrobin (102). In *Drosophila* cultured cell lines, depletion of centriole wall proteins, including SAS-4, enhances centriole loss (60). In mammalian cell lines, loss of the SAS-4 ortholog CPAP results in centriole fragmentation, though centriole loss has not been reported (104).

It is also possible that the compound microtubules themselves may be inherently more stable than singlet microtubules. It has been challenging to directly test this possibility, as isolation of centrioles and axonemes also results in the copurification of other associated proteins. Doublet microtubule assembly *in vitro* requires microtubule stabilization with paclitaxel and the slowly hydrolysable GTP analogue, GMPCPP, and thus it is experimentally challenging to directly test the stability of the microtubules themselves (105). In addition, *C. elegans* centrioles are stable and contain singlet microtubules with partial B-tubules (106), indicating that complete compound microtubules are not universally required for centriole stability.

### Tubulin post-translational modifications

Alpha- and beta-tubulin are highly post-translationally modified (107), and these modifications can alter the dynamics and stability of microtubules and the binding of microtubule-associated proteins. One of the earliest observations of centriole loss in cultured cell lines was made following the injection of the anti-glutamylation antibody GT335 (108). GT335 recognizes the initial branch point of monoglutamylation on alpha- and more weakly on beta-tubulin, which allows recognition of glutamate chains of any length (109). The centriole walls are highly glutamylated, and GT335 is routinely used to mark centrioles in immunofluorescence microscopy. Direct injection of GT335 antibody into mammalian cells results in centriole disintegration coupled to loss of the pericentriolar material (108). This finding suggests that centriole stability depends on either glutamylated tubulin, proteins that bind to glutamylated tubulin, or proteins whose binding is inhibited by the GT335 antibody.

*In vivo*, loss of glutamylation does not seem to result in complete centriole loss. Mice lacking the major writers for polyglutamylation, TTLL1 and TTLL7 (110, 111), can produce viable offspring (112, 113), in contrast to the embryonic lethality of mice that lack centrioles through knockout of CPAP or SASS6 (114, 115). Overexpression of CCP5, which removes polyglutamylation from microtubules, also does not impair centriole stability (116). In *Tetrahymena*, double knockouts of the polyglutamylation writers TTLL1 and TLL9 lead to defects in basal body maturation and basal body loss (117, 118). Moreover, injection of GT335 antibody into mammalian cells results in increased centriole fragmentation in G2/M due to enhanced microtubule dynamics at the onset of mitosis (119). Together, these experiments indicate that glutamylation may be involved in maintaining centriole and basal body stability under mechanical force.

Additional tubulin post-translational modifications (PTMs) can alter the dynamics and stability of microtubules and the binding of microtubule-associated proteins. These include acetylation, glycylation, and a cycle of detyrosination and tyrosination (107). *A priori*, these may affect centriole stability as well. While the effects of these other PTMs on centriole stability have not been studied directly, genetic manipulations that prevent these modifications do not result in organismal phenotypes that would indicate premature centriole loss. Mouse knockouts of the major tubulin acetyltransferase (ATAT1), glycylation writers (TTLL10 and TTLL8, and TTLL3), and detyrosination enzymes (VASH1 or VASH2, and MATCAP1 and MATCAP2) are viable (120, 121, 122, 123, 124, 125, 126, 127, 128). Glycylation is necessary in *Tetrahymena* to maintain basal body organization against forces from ciliary beating (129), indicating that glycylation is also involved in maintaining basal body stability under mechanical force. We note that the effects of these other modifications on centriole or basal body stability have not been studied directly in mammalian systems. There may be tissue-specific requirements for each, such that some post-translational modifications act to inhibit centriole loss in particular tissues. Redundancy of enzymes or post-translational modifications may also complicate these analyses, and targeted studies will be important to determine how these modifications affect centriole stability.

### Centriole length

In mammalian cell lines, fully mature centrioles reach approximately 500 nm in length (29). Centriole over-elongation has been observed in cancer cell lines (72) and plasma cells (130), where it is accompanied by the presence of broken microtubule walls and centriole fragmentation. In cancer cell lines, centriole fragments can drive centriole amplification (72). In cultured cell lines, loss of proteins such as RTTN, CEP295, CPAP, and CEP350 results in defects in both centriole length and integrity (101, 104, 32, 131). However, for CEP350 mutants, centriole fragmentation was due to loss of the inner scaffold protein WDR90, and not a direct consequence of centriole over-elongation. In these experiments, loss of CEP78 or OFD1 also results in centriole over-elongation, but no defects to the microtubule wall or fragmentation are observed (131). Similarly, overexpression of CPAP, cell cycle arrest, or overexpression of CEP120 results in elongated procentrioles with no concurrent centriole fragmentation or loss phenotype (29, 132, 133, 134). Thus, over-elongation alone is not sufficient to induce centriole fragmentation, and additional assembly defects result in a loss of integrity.

## Additional centrosome substructures

### Cartwheel

The cartwheel, formed of a hub and spokes arranged in nine-fold symmetry, is one of the first structures formed in the assembly of a procentriole. The cartwheel creates the nine-fold symmetry of the centriole, and the spokes attach to the centriolar microtubules through the pinhead (135). The major protein component of the cartwheel, SASS6, oligomerizes *in vitro* to form the cartwheel hub and central region of the spokes (16, 17). In human cell lines and *C. elegans*, SASS6/SAS-6 is essential for centriole formation, and thus it has been difficult in these systems to determine whether the cartwheel has an additional role in promoting centriole stability. Interestingly, the loss of centriole integrity due to the depletion of centriole lumen proteins can be partially rescued by the retention of SASS6 during prolonged mitotic arrest, suggesting that the cartwheel has an additional role in maintaining centriole stability (136).

Further evidence arises from mouse embryonic stem cells and *Drosophila,* where centrioles can form even when SASS6/DSAS-6 is absent (115, 137). Complete knockout of SASS6 from mouse embryonic stem cells results in loss of centriole integrity, with abnormally long or fragmented centrioles present (115). When these mutant cells are induced to differentiate into neural progenitor cells, centrioles are subsequently lost. Similarly, newly synthesized DSAS-6 protein is required in *Drosophila* cell lines to maintain centriole stability (60). These results indicate that the cartwheel is involved in maintaining centriole stability. However, the cartwheel is unlikely to be the sole determinant of centriole stability: SASS6 is lost from human centrioles in late mitosis (138, 139), but parental centrioles remain stable.

### A-C linker

At the proximal end of centrioles, A-C linkers connect the A-tubule of one triplet to the C-tubule of the adjacent triplet (Fig. 1). Long observed by electron microscopy, components of the A-C linker were only recently identified, and include the proteins CCDC77, WDR67, and MIIP (52, 79). Depletion of any of these three proteins leads to fragmentation of the centriole wall such that adjacent triplets splay out from each other (18). Co-depletion of CCDC77 and WDR67 exacerbated this effect. Thus, the A-C linker is required for maintaining centriole integrity by connecting adjacent triplet microtubules to each other. It is unclear whether centrioles lacking this structure are ultimately lost from cells.

### Centriole inner structure: the inner scaffold

In the central region of centrioles from mammals, *Chlamydomonas*, *Tetrahymena*, and *Paramecium*, a helical inner scaffold within the central centriole lumen connects adjacent microtubule triplets (Fig. 1) (21). Among the known proteins of the inner scaffold, including WDR90, CETN2, POC1A, POC1B, CCDC15, POC5, and FAM161A, several are involved in maintaining centriole integrity. POC5 recruits the augmin complex and the gamma-tubulin ring complex (γ-TuRC) to the centriole lumen, and both microtubule regulators are required for centriole integrity (136). These studies are summarized below, and together they demonstrate that the inner scaffold plays an important role in maintaining centriole integrity.

WDR90 directly links the inner scaffold to the centriolar microtubules. In human cells, WDR90 depletion leads to the loss of several inner scaffold proteins, accompanied by centriole fragmentation and splayed centriolar microtubules. Co-depletion of WDR90 and POC5 enhanced these defects (25). WDR90 localization to centrioles is dependent on CEP350, and loss of CEP350 also results in centriole fragmentation as a result of WDR90 mislocalization (131). CEP350 is regulated by the phosphatase PPP2R3C and the kinase MAP3K1. Overexpression of MAP3K1 also leads to centriole fragmentation and loss, though it is not clear whether this occurs through defects in CEP350 localization or function (140).

POC1 forms the inner junction between the A- and the B-tubules in *Tetrahymena* (23). While POC1 is not essential for basal body formation, loss of POC1 results in defects in inner scaffold stability, resulting in the structural instability of basal bodies because of forces imparted by ciliary beating (141). Vertebrates have two paralogs, POC1A and POC1B. In mammalian cells, depletion of either POC1A or POC1B results in loss or mislocalization of inner scaffold proteins and short centrioles with broken centriolar walls. Double knockout of both POC1A and POC1B resulted in an exacerbated phenotype, with reduction of centriole numbers in interphase and their loss during mitosis, indicating that POC1A and POC1B together are essential for centriole stability (142).

Centrin is a small EF-hand protein that localizes within the inner scaffold, as well as at the distal tip (79, 143). In human cells, centrin was reported to be involved in centriole formation, and thus it has been difficult to determine whether it may also have a role in maintaining centriole integrity (144). In *Tetrahymena*, Cen1, which is orthologous to human centrin 2, is required for both basal body formation and stability (145). *Tetrahymena* Cen2, which is orthologous to human centrin 3, is required for basal body stability (146). Both centrin proteins are also required for correct basal body orientation. Upon starvation, which inhibits new basal body formation, basal body number was reduced in cen2 mutants, indicating that cen2 is required for basal body maintenance. Similarly, centriole loss was also observed in cen1 mutant cells, indicating that centrin is important for centriole stability.

CCDC15 is required for the localization of POC1B and the distal end proteins SFI1/CETN2. Upon depletion of CCDC15, centrioles had structural integrity defects, with broken, wider, or shorter walls (22). Upon prolonged mitotic arrest, some cells lost centrin foci, indicating that the structural integrity defects in CCDC15-depleted cells result in either complete loss of centrioles or partial loss of the centriole distal end. It is unclear whether these fragmented centrioles are ultimately lost from cells or whether they may be retained.

Within the centriole lumen, the augmin complex and the gamma-Tubulin Ring Complex (γ-TuRC) stabilize centrioles (136). These microtubule nucleators are recruited by POC5. Loss of the augmin component HAUS6 resulted in the presence of short, broken, or incomplete centrioles. Upon prolonged mitotic arrest, cells lacking POC5, HAUS6, or the γ-TuRC component GCP4 lost their distal ends, indicating that these proteins also maintain centriole integrity (136). It is unclear whether these fragments are lost or retained in cells. Similarly, in *Tetrahymena*, loss of γ-tubulin results in both defects in forming new basal bodies as well as loss of existing basal bodies, indicating that γ-tubulin is important for maintaining centriole stability (147). However, in this study, it is unclear whether γ-tubulin might be required within the centriole lumen, in the PCM (below), or at other microtubule structures.

### Centriole inner structure: The *C. elegans* central tube

In the nematode *C. elegans*, a structure known as the central tube is critical for centriole stability (61). *C. elegans* centrioles, unlike the centrioles of other organisms, have singlet microtubules with doublet microtubule hooks and are short, approximately 150 nm in length (83, 106, 148). The cartwheel-equivalent, known as the inner tube, extends the entire length of the centriole (83, 148). A central tube is positioned between the inner tube and the centriolar microtubule walls. During centriole loss in oogenesis, the central tube is the first substructure to be removed from centrioles (61). Central tube loss is followed by centriole widening, centriolar microtubule fragmentation, and disassembly. Additional centriolar proteins are lost gradually afterwards, driven by microtubule and dynein-dependent movement toward the plasma membrane.

The central tube consists of SAS-1 and SSNA-1 (69, 61, 149). Neither SAS-1 nor SSNA-1 is required for centriole formation, but both are involved in maintaining centriole stability. Depletion or absence of SAS-1 results in premature centriole loss during oogenesis, as well as defects in centriole integrity and centriole loss in sperm and embryos (61, 149). SAS-1 recruits SSNA-1 to centrioles (149), and loss of SSNA-1 results in aberrant centriole fragmentation in embryos (69). These results indicate that SAS-1 and SSNA-1 are essential for centriole stability in *C. elegans*, and that the loss of one protein or centriole substructure can trigger centriole loss in cells. The human orthologs of SAS-1 and SSNA-1 (C2CD3 and SSNA1, respectively) localize to the distal regions of human centrioles (150). Depletion of C2CD3 or SSNA1 from human cell lines does not seem to result in centriole fragmentation (150), suggesting that additional structures found within human centrioles, such as the inner scaffold, provide additional support.

### Distal tip

Proteins at the centriole distal end include SFI1 and CETN2/3, the DISCO complex (OFD1, MNR, CEP90, and FOPNL), C2CD3, CBY1, and the distal cap complex (CPAP, CEP97, and CP110) (79, 143, 150, 151, 152, 153, 154, 155, 156). Among these, SFI1 has been demonstrated to be important for centriole integrity in human cell lines. SFI1 localizes, along with centrin, to a dot within the distal lumen of centrioles (143). SFI1 is not involved in centriole assembly, but mature centrioles in cells depleted of SFI1 are often structurally defective with broken or fragmented walls, indicating that SFI1 is essential for maintaining centriole structural integrity after formation (143). It is unclear whether these structurally defective centrioles are ultimately lost from cells.

### Pericentriolar material (PCM)

During the centriole duplication cycle, PCM is initially recruited to newly formed procentrioles in mitosis in a process known as centriole-to-centrosome conversion. CEP295/ANA1 is a proximal centriole protein important for centriole-to-centrosome conversion and centriole stability. Depletion of CEP295/ANA1 from mammalian or *Drosophila* cell lines results in the inability to recruit PCM components, including ASL/CEP152 and CEP192 (33, 157). Depletion or loss of CEP295 leads to centriole loss in cultured human cell lines (32, 33, 157), indicating that centriole-to-centrosome conversion may be essential for centriole stability. However, centriole instability following CEP295 loss may also be attributed to defects in inner scaffold assembly, as CEP295 is also crucial for centriole elongation and the recruitment of inner scaffold proteins POC5 and POC1B in cultured human cell lines (158).

The PCM is also important for centrosome loss during oogenesis in *Drosophila*. In this system, centrosome loss proceeds sequentially: the PCM is lost first, followed by the loss of centriole components. PCM loss is regulated by the kinase Polo/PLK1. Loss of Polo occurs concomitantly with the loss of PCM, and premature depletion of Polo results in premature centriole loss in oocytes. In *Drosophila* cell lines, depletion of Polo or simultaneous depletion of 4 different PCM proteins (ASL, CNN, D-PLP, and SPD2) leads to centriole loss (59). Remarkably, ectopically tethering Polo to centrosomes in oocytes prevented the loss of both PCM and centrioles. This led to the presence of functional centrosomes during meiosis, resulting in early embryonic arrest caused by chromosome segregation defects.

How does Polo maintain centriole stability? Ectopically tethering kinase-dead Polo to centrosomes did not completely prevent centriole loss, indicating that the kinase activity of Polo is likely required (59). Further work showed that Polo is required to maintain the proximal-end centriole wall protein ANA1/CEP295 (60). ANA1 depletion results in enhanced centriole loss in *Drosophila* cell lines or oocytes (60). Tethering ANA1 to centrosomes is sufficient to prevent their loss. Surprisingly, this effect was independent of PCM recruitment, demonstrating that ANA1 itself, rather than the PCM, is required for centriole stability (60). Supporting this conclusion, the stable centrioles formed in this situation could not act as microtubule organizing centers. Together, these results indicate that PCM loss can lead to the depletion of components critical for centriole stability (59, 60). We note that in many differentiated cell types, the PCM is relocated from centrosomes to other cellular positions (159, 160). Centriole structure has not been systematically studied in these systems, and it would be informative to determine whether they may be lost.

## Conclusion and outlook

Centrosome substructures are important regulators of centriole stability, paving the way for future mechanistic insights into centriole loss. The proteins and substructures reviewed here could serve as molecular markers to clarify and differentiate between models of centriole stability (Fig. 3). For instance, in an inherent stability model, it is essential to investigate how these proteins and structures may be modified during centriole loss. Are proteins post-translationally modified or altered during loss? Do substructures have altered morphology or positioning? Does the loss of integrity always lead to centriole loss?

To directly test models of active repair or constant renewal, it is critical to investigate the turnover kinetics of proteins at centrioles. While experiments using FRAP have examined turnover on short timescales (seconds to minutes), constant renewal likely operates over longer periods. In *Drosophila* cell lines, assays for centriole stability demonstrate that new protein exchange can take up to 8 days (59, 60). Techniques such as pulsed SILAC (74) can be valuable for determining the turnover rates of specific proteins over extended timescales. Pulsed SILAC could also help identify changes in turnover rates during centriole loss, which might accelerate during active repair. In an active repair model, it is critical to identify specific molecules that recognize and remove damaged proteins and replace them with new components. Depletion or mutation of these molecules may alter centriole loss kinetics.

In cases of targeted degradation, it is crucial to identify the molecules involved, such as autophagy adaptors or receptors. Understanding how these molecules recognize centrioles, possibly through association with centriolar satellites, is also important.

With recent advances in imaging and protein identification, additional proteins and substructures may also be implicated in centriole structural stability in the future. It will be important to determine whether these proteins are involved in centriole formation and/or whether they may be specifically involved in maintaining centriole stability. High-resolution imaging of centrioles in different tissues and cell types, as well as in patient samples, may also reveal additional contexts in which centrioles are lost. Understanding the mechanisms underlying centriole loss will pave the way for new techniques to manipulate centriole number and structure in cells, enabling precise control of this crucial organelle in development and disease. (161, 162, 163, 164, 165, 166, 167, 168, 169, 170, 171, 172, 173, 174)(155)(175, 176, 177, 178, 179, 180, 181, 182)

## Conflict of Interest

The authors declare that they do not have any conflicts of interest with the content of this article.
